# Factors associated with the use of antibiotics for children presenting with illnesses with fever and cough obtained from prescription and non-prescription sources: a cross-sectional study of data for 37 sub-Saharan African countries

**DOI:** 10.1186/s12889-024-18490-1

**Published:** 2024-04-19

**Authors:** Meklit Melaku Bezie, Zufan Alamrie Asmare, Hiwot Altaye Asebe, Afework Alemu Lombebo, Bezawit Melak Fentie, Angwach Abrham Asnake, Beminate Lemma Seifu

**Affiliations:** 1 Departmnet of Public Health Officer, College of Medicine and Health Sciences and Comprehensive Specialized Hospital, University of Gondar, Gondar, Ethiopia; 2 Department of Ophthalmology, School of Medicine and Health Science, Debre Tabor University, Debre Tabor, Ethiopia; 3 Department of Public Health, College of Medicine and Health Sciences, Samara University, Afar, Ethiopia; 4 School of Medicine, College of Health Science and Medicine, Wolaita Sodo University, Soddo, Ethiopia; 5 Department of General Midwifery, School of Midwifery, College of Medicine and Health Sciences, University of Gondar, Gondar, Ethiopia; 6 Department of Epidemiology and Biostatistics, School of Public Health, College of Health Sciences and Medicine, Wolaita Sodo University, Soddo, Ethiopia; 7 Department of Public Health, College of Medicine and Health Sciences, Samara University, Samara, Ethiopia

**Keywords:** Under-five children, Modified poisson regression analysis, Antibiotics, Fever, Cough, Sub-Saharan Africa

## Abstract

**Introduction:**

Fever and cough in under-five children are common and predominately self-limiting illnesses. Inappropriate prescribing of antibiotics in sub-Saharan Africa is a significant public health concern. However, prescription sources and use among children with fever or cough have not been explored. Therefore, we explored the factors associated with the use of antibiotics obtained from prescription and non-prescription sources for children with illnesses associated with fever and cough.

**Methods:**

A secondary data analysis was conducted based on the Demographic and Health Survey (DHS) data from 37 sub-Saharan African countries. A total weighted sample of 18,866 under-five children who had a fever/cough and took antibiotics were considered for this study. Given the hierarchical nature of DHS data and the use of antibiotics prescribed from the formal healthcare setting (> 10%), a multilevel modified poisson regression model was fitted. Deviance was used for model comparison and the model with the lowest deviance value was chosen as the best-fitted model. Variables with *p* ≤ 0.2 in the bivariable analysis were considered for the multivariable modified poisson regression model. In the multivariable multilevel modified poisson regression model, the Adjusted Prevalence Odds Ratio (APOR) with a 95% Confidence Interval (CI) and *p*-value < 0.05 were reported to declare a significant association with taking antibiotics for fever/cough prescribed from formal healthcare setting.

**Results:**

In sub-Saharan Africa, the proportion of use of antibiotics from informal healthcare setting for fever and cough among under-five children was 67.19% (95% CI: 66.51%, 67.85%). In the multilevel modified poisson regression analysis; residing in a rural area (APOR = 1.08, 95% CI: 1.04, 1.12), a child aged 36–47 months (APOR = 0.94, 95% CI: 0.90, 0.98), a child aged 48–59 months (APOR = 0.89, 95% CI: 0.84, 0.94), maternal primary education (APOR = 0.96, 95% CI: 0.93, 0.99), maternal secondary education (APOR = 0.95, 95% CI: 0.92, 0.99), belonged the middle household wealth status (APOR = 1.07, 95% CI: 1.02, 1.11), maternal exposure to news/electronic media (APR = 1.06, 95% CI: 1.02, 1.10), being from a household with 2 under-five children (APR = 0.94, 95% CI: 0.91, 0.97), being from a household with 3 under-five children (APR = 0.89, 95% CI: 0.85, 0.93), being from a household with 4 under-five children (APR = 0.90, 95% CI: 0.83, 0.98), and children of caregivers who were not involved in decision-making for their child health issues were significantly associated with taking antibiotics prescribed from formal healthcare setting for fever/cough among under-five children.

**Conclusion:**

Only two-thirds of the antibiotics used for children under five who had fever and cough were prescribed from formal healthcare setting. Our findings underscore the significance of addressing healthcare disparities, improving access to qualified healthcare providers, promoting maternal education, and empowering mothers in healthcare decision-making to ensure appropriate antibiotic use in this vulnerable population. Further research and interventions targeted at these factors are warranted to optimize antibiotic prescribing practices and promote responsible antibiotic use in the management of fever and cough in under-five children.

## Background

Fever and cough are common symptoms in under-five children and are often associated with self-limiting illness [1, 2]. However, the majority of cases are resolved on their own without the need for specific medical treatment, caregivers need to monitor their child's symptoms closely and seek medical attention if necessary [3]. Overuse and misuse of antibiotics are significant contributors to the emergence of antibiotic-resistant bacteria [4]. Antibiotic resistance is a serious global health concern as it can lead to infections that are difficult or impossible to treat. Following the introduction of antibiotics, significant reduction in bacterial disease-related mortality [5]. However, the misuse and overuse of antibiotics have led to the emergence of Antimicrobial Resistance (AMR) [6].

An estimated 5 million deaths in 2019 were attributed to bacterial antimicrobial resistance, according to the Global Burden of Bacterial Antimicrobial Resistance report [7]. Respiratory infections, the most common infectious syndrome, are responsible for over 1.5 million of these deaths [8]. These infections pose a significant challenge in sub-Saharan Africa (SSA), a region where, despite global advancements in healthcare, the mortality rate for children under five remains alarmingly high due to such prevalent infectious diseases [9, 10].

Empirical antibiotic therapy is indeed frequently used in SSA, where there is a high burden of infectious diseases and limited diagnostic capacity [11]. In this setting, clinicians prescribe antibiotics based on clinical judgment without waiting for diagnostic test results [12]. Such empirical therapy, while sometimes necessary, raises concerns about antibiotic overuse, a primary cause of AMR [13]. Understanding the determinants of antibiotic prescriptions from qualified sources of children under five who had a fever/cough can offer valuable insights, potentially guiding targeted interventions [14]. There are published studies on the source of antibiotic prescriptions that revealed that the majority of the antibiotics were not prescribed by formal health care professionals in the low-and middle-income countries such as SSA [15–17].

According to our literature review, there is no published study on whether the prescribed antibiotics for under-five children with fever/cough in SSA originated from qualified sources. Therefore, this study examined the factors associated with the use of antibiotics obtained from prescription and non-prescription sources for children with illnesses associated with fever and cough in SSA. The findings of this study would be crucial in reducing drug resistance and related complications.

## Methods and materials

### Data source

Secondary data analysis was conducted based on the most recent Demographic and Health Survey (DHS) data of 37 sub-Saharan African (SSA) countries that have data on the sources of prescriptions for antibiotics used for cough and fever among under-five children (Angola, Burkina Faso, Benin, Burundi, Dr Congo, Congo, Cote d’Ivoire, Cameroon, Ethiopia, Gabon, Ghana, Gambia, Guinea, Kenya, Comoros, Liberia, Lesotho, Madagascar, Mali, Mauritania, Malawi, Mozambique, Nigeria, Niger, Namibia, Rwanda, Sierra Leone, Senegal, Sao Tome, Swaziland, Chad, Togo, Tanzania, Uganda, South Africa, Zambia, and Zimbabwe). DHS is conducted every five years to generate updated health and health-related indicators. A two-stage stratified sampling technique was employed to recruit the samples with Enumeration Areas (EAs) and households as primary and secondary sampling units, respectively. The detailed methodology is available athttps://dhsprogram.com/Methodology/index.cfm. The data were extracted using the Kids Record dataset (KR file) after we obtained an authorization letter from the measure DHS program for data access. Mothers or caregivers of under-five children were asked about whether their children experienced fever and/or cough within 2 weeks within 2 weeks preceding the survey. Then, for those who experienced cough and/or fever were asked for the source of the prescription. A total weighted sample of 18,866 under-five children in SSA who took antibiotics for fever/cough were considered for this study (Table 1).

**Table 1:** Weighted sample size by country

Country	Weighted sample size	Percentage
Angola	565	2.99
Burkina Faso	878	4.65
Benin	375	1.99
Burundi	1124	5.96
Dr Congo	1498	7.94
Congo	1328	7.04
Cote d'Ivoire	448	2.38
Cameroon	172	0.91
Ethiopia	404	2.14
Gabon	579	3.07
Ghana	187	0.99
Gambia	434	2.3
Guinea	239	1.27
Kenya	305	1.62
Comoros	172	0.91
Liberia	423	2.24
Lesotho	104	0.55
Madagascar	385	2.04
Mali	258	1.37
Mauritania	115	0.61
Malawi	1157	6.13
Mozambique	343	1.82
Nigeria	1061	5.62
Niger	74	0.39
Namibia	496	2.63
Rwanda	598	3.17
Serra Leone	368	1.95
Senegal	645	3.42
Sao Tome	136	0.72
Swaizland	26	0.14
Chad	851	4.51
Togo	417	2.21
Tanzania	316	1.67
Uganda	1392	7.38
South Africa	206	1.09
Zambia	503	2.67
Zimbabwe	285	1.51
Total	18,866	100

### Measurement of the variables

The outcome variable was antibiotics taken for fever/cough prescribed from qualified sources. It was generated using three DHS questions such as "had fever/cough within 2 weeks?", "antibiotic taken for fever/cough?" and "source of prescription?". Then, we classified the source of prescription as prescribed from formal healthcare settings and prescribed from informal healthcare settings. Antibiotics prescribed from governmental hospitals, clinics, Non-governmental Organizations (NGOs), private hospitals, and public health sectors were classified as prescribed from formal healthcare settings, and shops, churches, traditional practitioners, drug sellers, friends, relatives, supermarkets, and others were classified as prescribed from informal healthcare settings [18]. Independent variables included in this study were the child's age, sex of the child, household wealth status, maternal age, maternal level of education, number of under-five children in the household, mothers' healthcare decision-making autonomy, media exposure, mother working status, delivery by cesarean section, duration of breastfeeding, residence and ever had vaccination.

### Data management and analysis

All the analyses reported in this study were based on the weighted data. STATA version 17 statistical software was used for the data management and analysis. As the proportion of antibiotics taken for fever/cough prescribed from formal health care settings among under-five children in SSA was 67%. In this case, using Odds Ratio (OR) as an effect measure could exaggerate the relationship between the dependent and independent variables. Therefore, we applied the multilevel modified poisson regression model to obtain the prevalence odds ratio.

We preferred this model because of the following reasons. For start, when the magnitude of the outcome variable is common, the odds ratio obtained using the binary logistic regression approach overestimates the strength of the relationship. Second, because the DHS data is hierarchical, mothers were nested within cluster/EA. As a result, our model considers data dependencies as well as the problem of overestimation.

The Likelihood Ratio (LR) test, Intra-class Correlation Coefficient (ICC), and Median Odds Ratio (MOR) were computed to measure the variation between clusters. The ICC quantifies the degree of heterogeneity between clusters (the proportion of the total observed individual variation of antibiotics taken for fever/cough prescribed from qualified sources among under-five children that is attributable to cluster variations).

$ICC= {\delta }^{2}/{(\partial }^{2}+ {\pi }^{2})$ [19], but MOR quantifies the variation or heterogeneity in outcomes between clusters and is defined as the median value of the odds ratio between the cluster at more likely to have antibiotics taken for fever/cough prescribed from formal health care settings and cluster at lower risk when randomly picking out two clusters (EAs) [20].

$$\text{MOR}=\exp\left(\sqrt{2\ast\partial2\ast0.6745}\right)\sim\text{MOR}=\exp\left(0.95\ast\partial\right).$$

$\partial^2$ indicates that cluster variance

Variables with a *p*-value < 0.2 in the bi-variable multilevel modified poisson regression analysis were considered for the multivariable analysis. Deviance was used to verify model fitness and a model with the lowest deviance was considered the best-fit model. Finally, the Adjusted Prevalence Odds Ratio (APOR) with its 95% confidence interval (CI) was reported, and variables with a *p*-value < 0.05 in the multivariable analysis.

## Ethical consideration

There was no need for ethical clearance as the researcher did not interact with respondents. The data used was obtained from the MEASURE DHS Program, and permission for data access was obtained from the Measure DHS program through an online request from http://www.dhsprogram.com.

## Results

### Descriptive characteristics of study participants

**Fig. 1:**
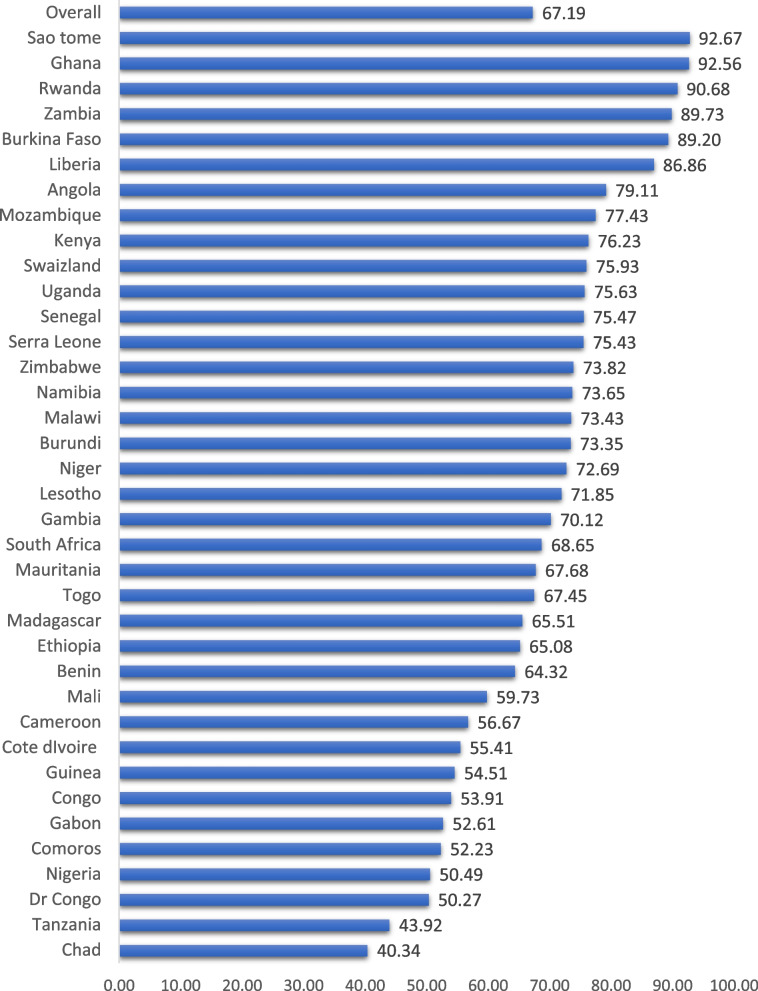
Proportion of under-five children who took antibiotics prescribed from informal sources in SSA

A total weighted sample of 18,866 under-five children in SSA who took antibiotics for fever/cough were included. Among them, 11,664 (61.83%) were residing in rural areas. About 9,618 (50.98%) were males and 2,899 (27.01%) were aged 12–23 months. Regarding household wealth status, 3,618 (19.18%) and 3,829 (20.29%) of the children belonged to the poorest and richest households, respectively. Among 18,866 under-five children who experienced fever and took antibiotics, about 7048 (37.36%) of the children's mothers attained a primary level of education (Table 2). The proportion of use of antibiotics prescribed from unqualified sources for under-five children who had a fever/cough and took antibiotics was 67.19% (95% CI: 66.51%, 67.85%). It ranged from 40.34% in Chad to 92.67% in Sao Tome (Fig. 1).

**Table 2:** Distribution of under-five children who had cough/fever and took antibiotics in SSA

Variables	Frequency	Percentage (%)
**Residence**		
Urban	7,202	38.17
Rural	11,664	61.83
**Child age (in months)**		
< 12	2,649	24.68
12—23	2,899	27.01
24—35	2,212	20.61
36 – 47	1,716	15.99
48 – 59	1,257	11.72
**Sex of child**		
Male	9,618	50.98
Female	9,248	49.02
**Maternal age (in years)**		
15–24	5,696	30.19
25–34	9,051	47.98
35–49	4,119	21.83
**Maternal educational status**		
No	5,251	27.83
Primary	7,048	37.36
Secondary	5,769	30.58
Higher	797	4.23
**Household wealth status**		
Poorest	3,619	19.18
Poorer	3,648	19.34
Middle	3,782	20.05
Richer	3,988	21.14
Richest	3,829	20.29
**Media exposure**		
No	5,483	29.06
Yes	13,383	70.94
**Number of under-five children**		
One	6,754	35.80
Two	7,250	38.43
Three	2,852	15.12
Four	871	4.62
Five and above	1,138	6.03
**Mothers employment status**		
Not working	5,933	31.49
Working	12,908	68.51
**Mode of delivery**		
SVD	17,614	93.36
Cesarean section	1,252	6.64
**Mothers decision-making autonomy**		
Mothers alone	2,904	15.40
Jointly with their partner/husband	5,730	30.37
Husband/partner alone	7,226	38.30
Someone else	3,006	15.93
**Ever had vaccination**		
No	561	10.30
Yes	4,882	89.70

### Multilevel-modified poisson regression results

The multilevel modified poisson regression model was the best-fitted model (deviance = 13,469) and had a smaller deviance value than the standard regression model (deviance = 13,502). In addition, the Likelihood Ratio (LR) test was statistically significant (*p* < 0.05), indicating that the advanced model was the best-fitted model for the data.

In the multivariable multilevel modified poisson regression analysis; residence, age of the child, mother's level of education, household wealth status, number of under-five children, and mother's healthcare decision-making autonomy were significantly associated with the use of antibiotics prescribed from formal health care setting for fever/cough among under-five children. The prevalence odds of the use of antibiotics prescribed from formal health care settings for fever and cough of under-five children were increased by 1.23 times (AOR = 1.08, 95% CI: 1.04, 1.12) compared to urban children. The prevalence odds of the use of antibiotics prescribed from formal health care setting for fever/cough among under-five children born to mothers whose husband and someone else decided on their child health issues were decreased by 10% (AOR = 0.90, 95% CI: 0.86, 0.94) and 5% (AOR = 0.95, 95% CI: 0.90, 0.99) compared to those who made their child health issues alone, respectively.

Children belonging to middle household wealth status had 1.23 times (AOR = 1.07, 95% CI: 1.02, 1,11) higher prevalence odds of taking antibiotics prescribed from formal health care setting for fever/cough compared to those belonging to the poorest households. The prevalence odds of taking antibiotics prescribed from formal health care settings among children in households that had two, three, and four under-five children were decreased by 6% (AOR = 0.94, 95% CI: 0.91, 0.97), 11% (AOR = 0.89, 95% CI: 0.85, 0.93) and 10% (AOR = 0.90, 95% CI: 0.83, 0.98) compared to having one under-five children, respectively.

Maternal exposure to news/electronic media increased the prevalence odds of taking antibiotics prescribed from formal health care settings for their children with fever/cough by 1.06 times (AOR = 1.06, 95% CI: 1.02, 1.10). The prevalence odds of taking antibiotics prescribed from formal health care setting for fever/cough among under-five children whose mothers had primary, and secondary education lowered by 4% (AOR = 0.96, 95% CI: 0.79, 0.93), and 5% (AOR = 0.95, 95% CI: 0.92, 0.99) compared to those whose mother had no formal education, respectively (Table 3).

**Table 3:** Factors associated with an antibiotic prescription from a formal health care setting for under-five children with fever/cough in SSA

Variables	Category	CPOR with 95% CI	APOR with 95% CI
Residence	Urban	1	1
	Rural	1.04 (1.01, 1.07)	1.08 (1.04, 1.12)^*^
Sex of child	Male	1	1
	Female	0.99 (0.97, 1.01)	1.01 (0.98, 1.03)
Mothers’ age (in years)	15–24	1	1
	25–34	1.01 (0.98, 1.03)	1.03 (0.99, 1.07)
	35–49	0.99 (0.96, 1.02)	1.03 (0.99, 1.07)
Child age (in months)	< 12	1	1
	12—23	1.01 (0.98, 1.05)	1.01 (0.97, 1.04)
	24—35	0.98 (0.94, 1.02)	0.97 (0.93, 1.01)
	36 – 47	0.95 (0.92, 0.99)	0.94 (0.90, 0.98)^**^
	48 – 59	0.91 (0.86, 0.95)	0.89 (0.84, 0.94)^**^
Mother's level of education	No	1	1
	Primary	0.97 (0.95, 1.00)	0.96 (0.93, 0.99)^**^
	Secondary	0.96 (0.94, 0.99)	0.95 (0.92, 0.99)^**^
	Higher	0.98 (0.93, 1.04)	0.99 (0.93, 1.08)
Household wealth status	Poorest	1	1
	Poorer	1.03 (0.99, 1.06)	1.02 (0.98, 1.06)
	Middle	1.07 (1.04, 1.11)	1.07 (1.02, 1.11)^*^
	Richer	1.02 (0.98, 1.06)	1.02 (0.97, 1.08)
	Richest	1.04 (1.00, 1.07)	1.03 (0.98, 1.09)
Media exposure	No	1	1
	Yes	1.07 (1.04, 1.10)	1.06 (1.02, 1.10)^*^
Number of under-five children in the household	One	1	1
	Two	0.95 (0.93, 0.98)	0.94 (0.91, 0.97)^**^
	Three	0.91 (0.88, 0.94)	0.89 (0.85, 0.93)^**^
	Four	0.90 (0.85, 0.96)	0.90 (0.83, 0.98)^*^
	Five and above	0.98 (0.93, 1.03)	0.95 (0.89, 1.02)
Mode of delivery	SVD	1	1
	CS	1.05 (1.01, 1.10)	1.03 (0.97, 1.09)
Mothers’ healthcare decision-making autonomy	Mothers alone	1	1
	Jointly with their partner/husband	0.99 (0.96, 1.02)	0.96 (0.92, 1.01)
	Husband/partner alone	0.91 (0.88, 0.95)	0.90 (0.86, 0.94)^*^
	Someone else	0.95 (0.92, 0.98)	0.95 (0.90, 0.99)^*^

^*^CPOR Crude Prevalence Odds Ratio, *APOR* Adjusted Prevalence Odds Ratio, *CI* Confidence Interval, ^*^: *p* < 0.05, ^**^: *p* < 0.01

## Discussion

In this study, the proportion of use of antibiotics prescribed from a formal health care setting for under-five children with fever and cough in SSA was found to be 67.19% (95% CI: 66.51%, 67.85%). In the multilevel modified poisson regression analysis; residence, media exposure, wealth status, number of under-five children in the household, maternal educational status, and women's autonomy in making healthcare decisions were significantly associated with the use of antibiotics prescribed from the formal healthcare setting.

This study showed that only two-thirds of under-five children with cough and fever took antibiotics prescribed by qualified healthcare settings in SSA. It was consistent with a previously published study [21], the limited availability and accessibility of healthcare services, along with limited trained healthcare professionals, are significant challenges faced by many sub-Saharan African countries [22]. Another reason can be due to the extreme poverty in SSA could limit people from visiting formal healthcare settings to obtain prescribed antibiotics as out-of-pocket expenses for diagnostic investigations and related costs are not affordable [23–25]. In addition, the cost of healthcare is high in many African nations, which makes people search for less expensive options. Because pharmacies are more cost-effective than certified healthcare providers, people buy antibiotics for less money [26].

Maternal educational status was significantly associated with taking antibiotics prescribed from the formal healthcare setting. This could be because education enhances mothers' knowledge towards healthcare-seeking behaviour for childhood illness and treatment [27]. This could boost their confidence to buy antibiotics for treating their children with cough and fever without visiting the health care facilities [28]. Acute febrile illness and cough among under-five children are common and self-limiting, and usually mothers or care-givers buy and give empirical therapy to their children.

Children living in rural areas had higher odds of using antibiotics prescribed from formal healthcare settings. It could be due to compared to urban residents, rural residents are not familiar and lack of confidence with the use of antibiotics without prescription from qualified healthcare professional [29, 30]. In addition, mothers or care-givers in rural areas may not be knowledgeable about their children medical needs or may not understand which antibiotics are used to treat diseases associated with cough and fever [31]. As a result, they may rely on advice from qualified health professionals, Secondly, the availability of pharmacies is higher in cities than in rural areas, which leads to the unsupervised acquisition of antibiotics without appropriate medical advice [32]. Together, these factors lessen the need for licensed medical professionals to prescribe antibiotics in urban settings. This research raises the possibility that rural and urban areas have different access to trained medical professionals, which could affect the recommendation and use of antibiotics for treating fever and cough in children under five.

We found that household wealth status significantly associated with the source of prescription for antibiotics for children with cough and fever. Children belonged to the middle household wealth had increased odds of using antibiotics prescribed from formal healthcare settings. This could be due to the fact that wealth status influences mother’s healthcare-seeking behavior and the likelihood of using antibiotics prescribed by qualified sources [33, 34]. Mothers with higher wealth status often have better access to healthcare services, including qualified healthcare providers who can accurately diagnose conditions and prescribe appropriate treatments, including antibiotics when necessary [35].

Efforts to improve access to healthcare services, particularly for marginalized populations, and to provide education on appropriate antibiotic use are essential for addressing disparities in antibiotic utilization and combating antibiotic resistance across all socio-economic groups.

Another significant predictor was the number of under-five children in the household. The higher the number of under-five children in the household the lower the odds of taking antibiotics prescribed from a formal healthcare setting for fever/cough compared to children in households with only one under-five child. This is because households with multiple children typically have greater experience with common childhood illnesses and treatment [36]. Therefore, they may self-diagnose and provide antibiotics without a prescription from formal healthcare. Besides, families with a higher number of under-five children might be struggling financially and more likely to look for more convenient and reasonably priced ways to get antibiotics [37].

Media exposure increases the likelihood of using antibiotics for fever/cough prescribed in formal healthcare settings. It is consistent with study findings reported in Bangladesh [38]. This could be because the media is a powerful tool in disseminating health-related information and raising awareness of available healthcare resources. Furthermore, the mother's healthcare decision-making autonomy was found a significant factor. Healthcare decisions for a child's health issue made by their father or someone else decrease the odds of receiving antibiotics prescribed from a formal healthcare setting for fever/cough. This highlights the importance of maternal autonomy in healthcare decision-making and its potential impact on antibiotic utilization patterns. Additionally, previous studies have indicated that lower maternal autonomy is linked to a reduced likelihood of seeking treatment.

### Strengths and limitations

Our study has notable strengths, including a large sample size encompassing 37 countries in the SSA region. In addition, we used a robust statistical approach to obtain the prevalence odds ratio to prevent the overestimation of the association between variables. However, it is important to acknowledge several limitations. Firstly, the cross-sectional design of the study restricts our ability to establish causal relationships between variables. Secondly, our classification of antibiotic sources was limited to formal and informal healthcare settings, and future studies could explore the associations between different factors and various sources of antibiotics. Further research is necessary to provide a comprehensive understanding of prescription patterns for antibiotics in other common childhood illnesses such as diarrhea, Acute Respiratory Infection (ARI), and others.

### Conclusion

The study has shown that the majority of the antibiotics used for fever and cough in under-five children were prescribed from formal healthcare settings. Overall, our study provides valuable insights into the factors associated with use of antibiotics prescribed from formal healthcare settings for fever and cough in under-five children. The findings underscore the significance of addressing healthcare disparities, improving access to qualified healthcare providers, promoting maternal education, and empowering mothers in healthcare decision-making to ensure appropriate antibiotic use in this vulnerable population. Further research and interventions targeted at these factors are warranted to optimize antibiotic prescribing practices and promote responsible antibiotic use in the management of fever and cough in under-five children.

## Data Availability

No datasets were generated or analysed during the current study.

## Abbreviations

APR: Adjusted prevalence ratio
CI: Confidence interval
DHS: Demographic health survey
EAs: Enumeration areas
LLR: Log likelihood ratio
LR: Likelihood ratio
SSA: Sub-Saharan Africa
WHO: World Health Organizations

## References

[1] Their, A.S.P.I., A.A. Lives, and A.F.A.F.O. All. Managing the child with a fever. Practitioner. 2015;259(1784):17–21. 26514056

[2] Adiele JA (2019). Knowledge, perception and health-seeking behaviour relating to childhood diarrhoea among mothers of under-five children in Eni-Osa community Ibadan, Oyo State, Nigeria.

[3] Tan T, Little P, Stokes T (2008). Antibiotic prescribing for self limiting respiratory tract infections in primary care: summary of NICE guidance. BMJ.

[4] Chokshi A (2019). Global contributors to antibiotic resistance. J Glob Infect Dis.

[5] Adedeji W (2016). The treasure called antibiotics. Ann Ib Postgrad Med.

[6] Organization, W.H (2021). Global antimicrobial resistance and use surveillance system (GLASS) report: 2021.

[7] Aljeldah MM (2022). Antimicrobial resistance and its spread is a global threat. Antibiotics.

[8] Marano N, Ahmed JA. Acute respiratory infection. Health in humanitarian emergencies: principles and practice for public health and healthcare practitioners. Cambridge: Cambridge University Press; 2018. p. 295–309.

[9] Acheampong M (2019). Priority setting towards achieving under-five mortality target in Africa in context of sustainable development goals: an ordinary least squares (OLS) analysis. Glob Health Res Policy.

[10] Yaya S (2019). Decomposing the rural-urban gap in the factors of under-five mortality in sub-Saharan Africa? Evidence from 35 countries. BMC Public Health.

[11] Kariuki S, Dougan G (2014). Antibacterial resistance in sub-Saharan Africa: an underestimated emergency. Ann N Y Acad Sci.

[12] Belachew SA, Hall L, Selvey LA (2021). Non-prescription dispensing of antibiotic agents among community drug retail outlets in Sub-Saharan African countries: a systematic review and meta-analysis. Antimicrob Resist Infect Control.

[13] Zargar A (2019). Overcoming the challenges of cancer drug resistance through bacterial-mediated therapy. Chronic Dis Transl Med.

[14] McKay R (2016). Systematic review of factors associated with antibiotic prescribing for respiratory tract infections. Antimicrob Agents Chemother.

[15] Minzi O, Manyilizu V (2013). Application of basic pharmacology and dispensing practice of antibiotics in accredited drug-dispensing outlets in Tanzania. Drug Healthc Patient Saf.

[16] Abula T, Worku A, Thomas K (2006). Assessment of the dispensing practices of drug retail outlets in selected towns, north west Ethiopia. Ethiop Med J.

[17] Gebrekirstos NH (2017). Non-prescribed antimicrobial use and associated factors among customers in drug retail outlet in Central Zone of Tigray, northern Ethiopia: a cross-sectional study. Antimicrob Resist Infect Control.

[18] Samir N (2021). Antibiotic use for febrile illness among under-5 children in Bangladesh: a nationally representative sample survey. Antibiotics.

[19] Rodriguez G, Elo I (2003). Intra-class correlation in random-effects models for binary data. Stand Genomic Sci.

[20] Merlo J (2006). A brief conceptual tutorial of multilevel analysis in social epidemiology: using measures of clustering in multilevel logistic regression to investigate contextual phenomena. J Epidemiol Community Health.

[21] Hossain MS (2023). Antibiotic prescription from qualified sources for children with fever/cough: cross-sectional study from 59 low-and middle-income countries. EClinicalMedicine.

[22] Gumede DM, Taylor M, Kvalsvig JD (2021). Engaging future healthcare professionals for rural health services in South Africa: students, graduates and managers perceptions. BMC Health Serv Res.

[23] Leive A, Xu K (2008). Coping with out-of-pocket health payments: empirical evidence from 15 African countries. Bull World Health Organ.

[24] Adebisi YA (2022). Revisiting the issue of access to medicines in Africa: challenges and recommendations. Public Health Challenges.

[25] Assefa Y (2017). Access to medicines and hepatitis C in Africa: can tiered pricing and voluntary licencing assure universal access, health equity and fairness?. Glob Health.

[26] Dalton K, Byrne S (2017). Role of the pharmacist in reducing healthcare costs: current insights. Integr Pharm Res Pract.

[27] Bosley H (2018). A systematic review to explore influences on parental attitudes towards antibiotic prescribing in children. J Clin Nurs.

[28] Alhomoud F (2017). Self-medication and self-prescription with antibiotics in the Middle East—do they really happen? A systematic review of the prevalence, possible reasons, and outcomes. Int J Infect Dis.

[29] Selvaraj K, Kumar SG, Ramalingam A (2014). Prevalence of self-medication practices and its associated factors in Urban Puducherry, India. Perspect Clin Res.

[30] Aqeel T (2014). Prevalence of self-medication among urban and rural population of Islamabad, Pakistan. Trop J Pharm Res.

[31] Sisay S, Endalew G, Hadgu G (2015). Assessment of mothers/care givers health care seeking behavior for childhood illness in rural Ensaro District, north Shoa zone, Amhara region, Ethiopia 2014. Glob J Life Sci Biol Res.

[32] Law MR (2013). The geographic accessibility of pharmacies in Nova Scotia. Can Pharm J.

[33] Khare S (2022). Antibiotic Use and Resistance in Under Five Children in Rural Central India: Implications of Caregivers’ Healthcare-Seeking Behaviour and Informal Healthcare Providers’ Practices.

[34] Geda NR (2021). Disparities in mothers’ healthcare seeking behavior for common childhood morbidities in Ethiopia: based on nationally representative data. BMC Health Serv Res.

[35] Escarce JJ, Kapur K. Access to and quality of health care, vol. 2006. Washington: National Academies Press; 2006.

[36] Colvin CJ (2013). Understanding careseeking for child illness in sub-Saharan Africa: a systematic review and conceptual framework based on qualitative research of household recognition and response to child diarrhoea, pneumonia and malaria. Soc Sci Med.

[37] Le Doare K (2015). Improving antibiotic prescribing for children in the resource-poor setting. Br J Clin Pharmacol.

[38] Nahar P (2020). What contributes to inappropriate antibiotic dispensing among qualified and unqualified healthcare providers in Bangladesh? A qualitative study. BMC Health Serv Res.

